# Publisher Correction: How morphological development can guide evolution

**DOI:** 10.1038/s41598-018-33706-2

**Published:** 2018-10-25

**Authors:** Sam Kriegman, Nick Cheney, Josh Bongard

**Affiliations:** University of Vermont, Department of Computer Science, Burlington, VT USA

Correction to: *Scientific Reports* 10.1038/s41598-018-31868-7, published online 17 September 2018

The original version of this Article contained errors. In the legend of Figure 3,

“Fitness of just the final, evolved populations (at generation 10000) are plotted in.”

now reads:

“Fitness of just the final, evolved populations (at generation 10000) are plotted in **B**.”

In the Results section, the symbols *ℓ* and *ℓ*^∗^ were not written in math font in the sentence “The endpoint parameters are denoted by asterisks: the controller develops from *ϕ* to *ϕ*^∗^, the morphology develops from *ℓ* to *ℓ*^∗^”.

In the legend of Figure 4, the symbol Φ was inadvertently italicised. As a result,

“Developmental windows (i.e. the total lifetime developmental change) for morphology, *W*_*L*_ (see Equation 5), and controller, *W*_*Φ*_ (see Equation 6), alongside fitness *F*.”

now reads:

“Developmental windows (i.e. the total lifetime developmental change) for morphology, *W*_*L*_ (see Equation 5), and controller, *W*_Φ_ (see Equation 6), alongside fitness *F*.”

and:

“(**B**) Median fitness as a function of morphology and controller development windows (*W*_*L*_, *W*_*Φ*_), for all Evo-Devo designs evaluated.”

now reads:

“(**B**) Median fitness as a function of morphology and controller development windows (*W*_*L*_, *W*_Φ_), for all Evo-Devo designs evaluated.”

In the Methods section under the subheading ‘Developmental windows’,

“The controller development window, *W*_*ϕ*_, is the sum of the absolute difference of starting and final phase offsets across the robot’s 48 voxels, divided by the total amount of possible controller development.”

now reads:

“The controller development window, *W*_Φ_, is the sum of the absolute difference of starting and final phase offsets across the robot’s 48 voxels, divided by the total amount of possible controller development.”

This section also contained errors in equations 5 and 6, where:

5 $${W}_{L}=\frac{1}{48(1.5)}\sum _{K=1}^{48}\,|{\ell }_{k}^{\ast }-{\ell }_{k}|$$

now reads:

5 $${W}_{L}=\frac{1}{48(1.5)}\sum _{k=1}^{48}\,|{\ell }_{k}^{\ast }-{\ell }_{k}|$$

and:

6 $${W}_{{\rm{\Phi }}}=\frac{1}{48\pi }\sum _{K=1}^{48}\,|{\phi }_{k}^{\ast }-{\phi }_{k}|$$

now reads:

6 $${W}_{{\rm{\Phi }}}=\frac{1}{48\pi }\sum _{k=1}^{48}\,|{\phi }_{k}^{\ast }-{\phi }_{k}|$$

Finally, in the Data Availability section, “github.com/skriegman/how-devo-can-guide-evo” was not correctly hyperlinked.

These errors have now been corrected in the HTML and PDF versions of this Article.

