# Supplementary material for: How morphological development can guide evolution
Source: Sci Rep. 2018 Sep 17;8:13934. doi: 10.1038/s41598-018-31868-7 (PMC6141532; doi:10.1038/s41598-018-31868-7)
Supplement: Supplementary file 2 — Supplementary Information [file 41598_2018_31868_MOESM2_ESM.pdf]

Supplementary information for:

How morphological development can guide evolution

Sam Kriegman*, Nick Cheney, and Josh Bongard

University of Vermont, Department of Computer Science, Burlington, VT, USA

*sam.kriegman@uvm.edu

This PDF file includes the following:

Video S1 (link)
Discussion: embodiment
Methods: rigid-bodied robots
Methods: mutations for soft robots
References
Figures S1 – S5

Supplementary Video

Supplementary Video S1.

This video (youtu.be/Ee2sU-AZWC4) provides a high-level overview of the results reported in this paper.

Supplementary Discussion

Embodiment.

We consider an agent to be embodied if its output affects its input. This relationship may be represented by the simple update rule $\ell_{t+1} = f(\ell_t, \phi)$, where ℓ_t denotes the morphology of an agent at time t, and ϕ denotes its control policy. In a disembodied system, changes to the morphology are not directly constrained by its current state; the update rule becomes: $\ell_{t+1} = f(\phi)$.

Once a round robot begins rolling, its control policy cannot instantaneously force the system to go in the other direction, since momentum will tend to preserve forward movement. This has the effect of reducing selection pressure on the controller, since fewer variations are deleterious. This allows evolution to continue climbing fitness gradients by mutating the controllers within these permissive body plans.

This might also be possible in disembodied agents if other dimensions of the system can be changed by some search process such as to facilitate the search for ϕ.

Supplementary Methods

Rigid-bodied robots.

Rigid-bodied robots and their environment were simulated using Pyrosim (ccappelle.github.io/pyrosim). The robot is a quadruped with a large, spherical abdomen; each leg is attached by a single degree-of-freedom hinge joint.

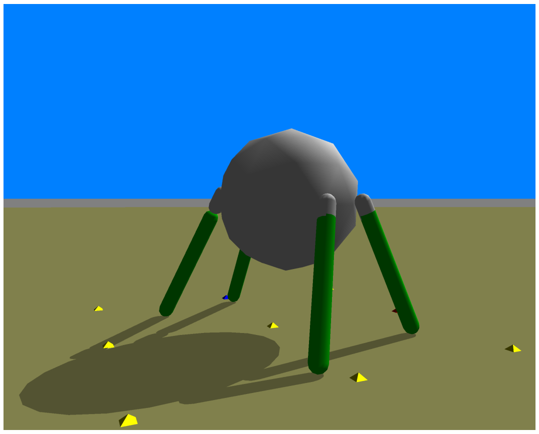

Morphological development was approximated in rigid bodies using linear actuators to slowly lengthen or shorten the length of each leg, from an evolved starting value (between 0 and 1) to an evolved final value (between 0 and 1). The controller is a simple neural net: two central pattern generators are fully connected to four motor neurons, each of which innervate a separate hinge joint. Controller development was approximated in neural networks through ballistic change to each synaptic weight: As the simulation proceeds, each weight develops linearly, from an evolved starting value (between -1 and +1) to an evolved final value (between -1 and +1).

The genotype spans two arrays: one for initial and final synaptic weights (controller), and another for initial and final leg lengths (morphology). Mutations affect, on average, a single element in each array. Apart from the genotype and its mutations, the evolutionary algorithm is identical to that of the soft robots. However, the task environment now consists of a sloped floor, declined toward a light source; and performance is measured by the average light intensity recorded by a light sensor embedded in the center of the agent's abdomen, according to the inverse square law of light propagation, at each time step in its life. Occlusion of the light caused by interference of the robot's own body parts was not simulated.

The results are presented below in Supplementary Fig. S5.

Mutations for soft robots.

The following derivation shows that there is a negligible difference in the mutations produced by the Evo and Evo-Devo treatments, in terms of the number of voxels modified (Fig. S4).

Each voxel cell of a soft robot has its own material properties that can be changed by the evolutionary algorithm. Evo voxels have two material properties: (1) resting length and (2) phase offset. Evo-Devo voxels have four material properties: (1) initial resting length, (2) final resting length, (3) initial phase offset, and (4) final phase offset.

Mutations are applied by first choosing which material properties to mutate, and then choosing, separately for each property, which voxels to modify. For each of the n material properties, we select it with independent probability $p = 1/n$. If none are selected, we randomly choose one. This occurs with probability $(1-p)^n$. Hence the number of selected material properties for mutation is a random variable S which follows a truncated binomial distribution,

$$\Pr(S = s \mid n) = \begin{cases} 0 & \text{for } s = 0 \\ np(1-p)^{n-1} + (1-p)^n & \text{for } s = 1 \\ \binom{n}{s} p^s (1-p)^{n-s} & \text{for } s > 1 \end{cases} \tag{1}$$

The expected number of selected material properties is then:

$$\begin{aligned} \mathbb{E}(S) &= np(1-p)^{n-1} + (1-p)^n + \sum_{s=2}^{n} s \binom{n}{s} p^s (1-p)^{n-s} & (2) \\ &= (1-p)^n + \sum_{s=1}^{n} s \binom{n}{s} p^s (1-p)^{n-s} & (3) \\ &= (1-p)^n + np & (4) \\ &= (1-p)^n + 1. & (5) \end{aligned}$$

For a selected material property, each voxel is mutated independently with probability λ, a hyperparameter we call the **mutation rate**. The expected *number* of genotype elements mutated given K total voxels is thus:

$$\delta_{\text{gene}} = \lambda K \cdot \mathbb{E}(S). \tag{6}$$

Dividing by the length of the genome, nK, we get the expected *proportion* of genotype elements mutated:

$$\pi_{\text{gene}} = \lambda / n \cdot \mathbb{E}(S). \tag{7}$$

Note that imposing bilateral symmetry does not change these expected values.

We have $K = 48$ total voxels, and $n = \{2, 4\}$ material properties for our two main experimental treatments {Evo, Evo-Devo}, respectively. The expected difference between a robot and its offspring, in terms of genotype elements, is summarized in the following table.

	$n = 2$	$n = 4$
δ_{gene}	60λ	63.8175λ
π_{gene}	0.625λ	0.3291λ

However, because multiple material properties can be mutated within a single voxel, the expected number of voxels mutated is lower than the expected number of genotype elements mutated. To calculate the average number of voxels mutated we need to consider a hierarchy of binomial distributions.

Given that S material properties were selected for mutation, the number of material properties mutated within a single voxel, M follows a binomial distribution,

$$\Pr(M = m \mid S, \lambda) = \binom{S}{m} \lambda^m (1-\lambda)^{S-m}. \tag{8}$$

For brevity, let's denote the probability that at least one mutation occurs within the voxel as θ,

$$\theta = \Pr(M > 0 \mid S, \lambda) = 1 - (1-\lambda)^S. \tag{9}$$

Then the number of voxels mutated, V, across a total of K voxels and S selected material properties, also follows a binomial distribution:

$$\Pr(V = v \mid S, K, \lambda, n) = \binom{K}{v} \theta^v (1-\theta)^{K-v}. \tag{10}$$

And the expected number of voxels mutated (out of K total) is

$$\delta_{\text{vox}} = \mathbb{E}(V \mid K, \lambda, n) \tag{11}$$
$$= \mathbb{E}_S \mathbb{E}_V (V \mid S, K, \lambda, n) \tag{12}$$
$$= \mathbb{E}_S (K\theta \mid S, K, \lambda, n) \tag{13}$$
$$= K \left\{ 1 - \mathbb{E}_S \left[(1-\lambda)^S \mid \lambda, n \right] \right\} \tag{14}$$
$$= K \left\{ 1 - \left[(1-\lambda)(1-p)^n + \sum_{s=1}^{n} (1-\lambda)^s \binom{n}{s} p^s (1-p)^{n-s} \right] \right\} \tag{15}$$
$$= K \left\{ 1 - (1-p)^n \left[\left(\frac{\lambda p - 1}{p-1} \right)^n - \lambda \right] \right\} \tag{16}$$

There is an extremely tight bound on the proportion of voxels mutated, $\pi_{\text{vox}} = \delta_{\text{vox}}/K$, for any $n > 1$ (Fig. S4). Thus mutations in Evo ($n = 2$) and Evo-Devo ($n = 4$) have practically the same impact in terms of the number of voxels modified. For completeness, the following table displays δ_{vox} for the specific values of λ considered by our hyperparameter sweep ($K = 48$) (Fig. S3).

	λ								
		1/48	2/48	4/48	8/48	16/48	24/48	32/48	48/48
n	2	1.25	2.48	4.92	9.67	18.67	27	34.67	48
	4	1.3	2.6	5.14	10.05	19.17	27.46	34.98	48

Supplementary References

1. Ancel, L. W. Undermining the Baldwin expediting effect: does phenotypic plasticity accelerate evolution? *Theor. population biology* **58**, 307–319 (2000).

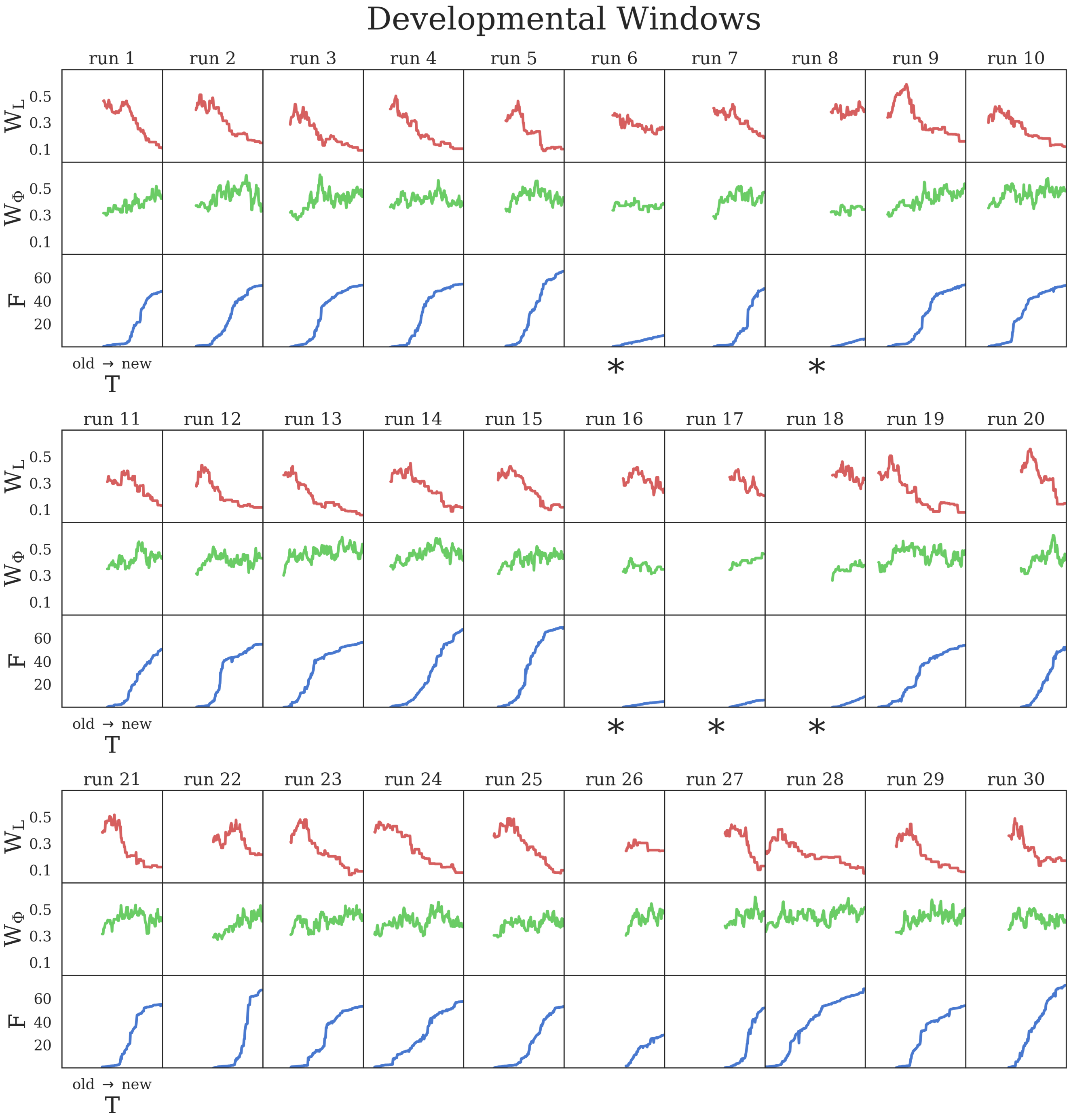

Figure S1. Evolutionary change during 30 Evo-Devo trials. The amount of morphological development, W_L (see Equation 3), controller development, W_Φ (see Equation 4), and fitness, F, for the lineages of the 30 Evo-Devo run champions. Evolutionary time, T, moves from the oldest ancestor (left) to the run champion (right). A general trend emerges wherein lineages initially increase their morphological development in T (rising red curves) and subsequently decrease morphological development to almost zero (falling red curves). Five of the 30 evolutionary trials, annotated by $*$, fell into a local optima.

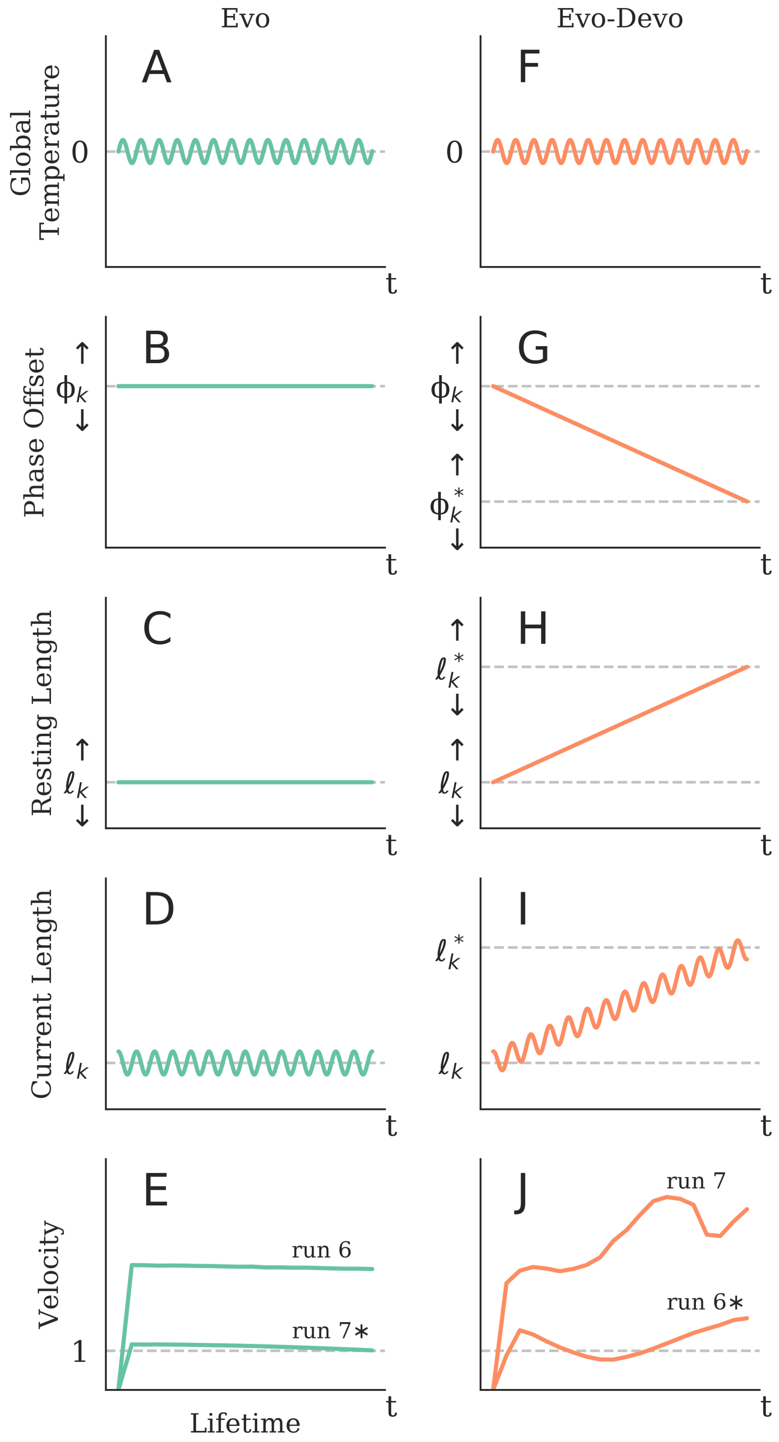

Figure S2. Experimental treatments. The phase of an oscillating global temperature (A, F) is offset in the k-th voxel by a linear function from ϕ_k to ϕ_k^* (B, G). The resting length of the k-th voxel is a linear function from ℓ_k to ℓ_k^* (C, H). For Evo, there is no development, so $\phi_k = \phi_k^*$ and $\ell_k = \ell_k^*$. The offset actuation is added on top of the resting length to give the current length of the k-th voxel (D, I). These example voxel-level changes occur across ontogenetic time (t), independently in each of the 48 voxels, and together interact with the environment to generate robot-level velocity (E, J). To see this, we averaged displacement across intervals of two actuation cycles (0.5 sec) and plotted the smoothed velocities for two Evo-Devo run champions with minimal canalization (J) alongside their non-developmental counterparts (E). Also note that the frequency of actuation is plotted here at 1.4 Hz; but, in our experiments, we used a frequency of 4 Hz.

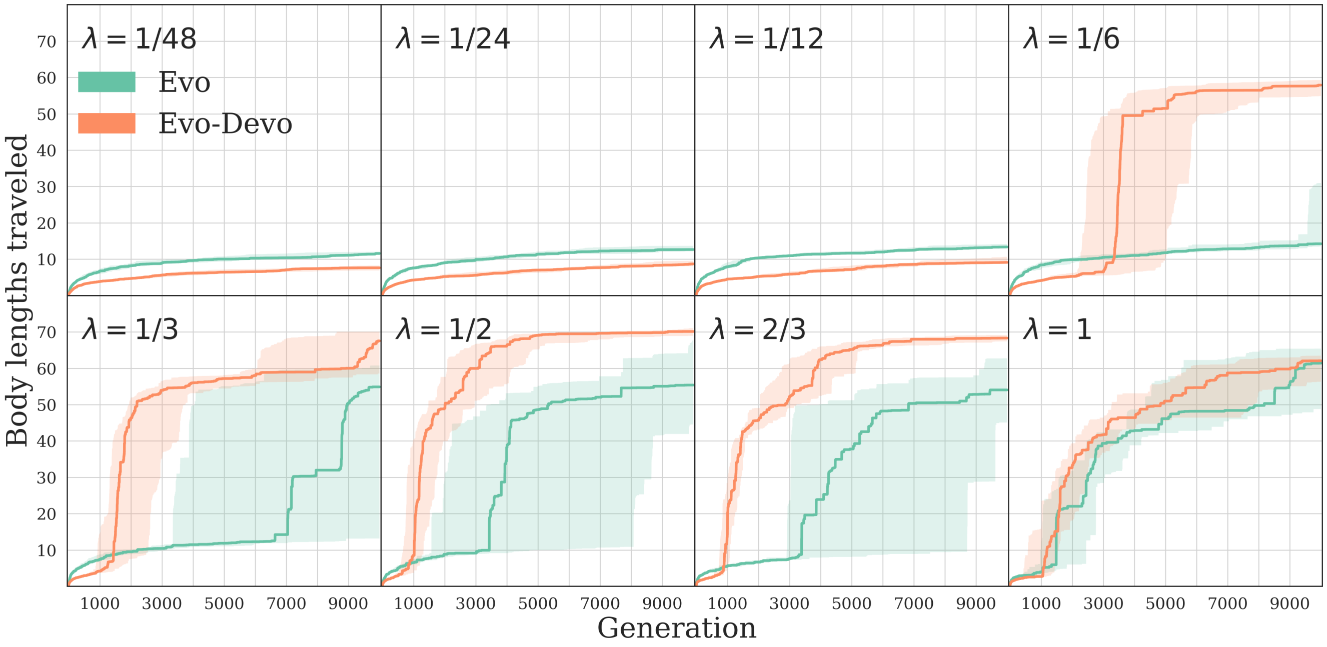

Figure S3. **Mutation rate sweep.** Median fitness (with 95% bootstrapped confidence intervals) under various mutation rates, λ, a hyperparameter defined in Supplementary Methods which affects the probability a voxel is mutated. In the main experiment of this paper, the mutation rate is evolved for each voxel independently, and is constantly changing. In this mutation rate sweep, λ is held uniform across all voxels. There were two observed basins of attraction in terms of fitness: a slower design that trots/gallops 5-15 body-lengths during the evaluation period, and a faster design type that rolls at 50-70 body-lengths. Although higher mutation rates facilitate the discovery of the superior phenotype, once found, lower mutation rates tend to produce more refined and faster robots. Without development, the search space has a single spike of high fitness. One can not do better than random search in such a space. When $\lambda = 1$, optimizing Evo morphologies reduces to random search, and this is the only mutation rate where Evo does not require significantly more generations than Evo-Devo to find the faster design type. This can be observed for $\lambda \in \{1/6, 1/3, 1/2, 2/3, 1\}$, by comparing the generation at which the slopes of the fitness curves increase dramatically. However, the best two treatments (Evo-Devo at $\lambda = 1/2$ and $\lambda = 2/3$), as measured by the highest median speed at the end of optimization, have development, and the robots they produced are significantly faster than those produced by random search (Evo with the highest mutation rate) $(p < 0.01)$.

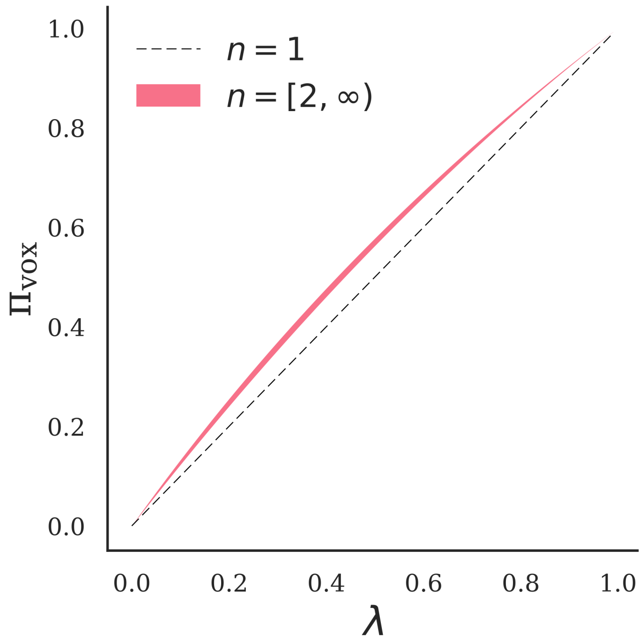

Figure S4. Mutational impact. The expected proportion of voxels modified, π_{vox}, where n is the number of material properties that can be mutated, and λ is the mutation rate. A derivation is provided in Supplementary Methods. Regardless of n, when $\lambda = 1$, every voxel must be mutated, and when $\lambda = 0$, no voxels can be mutated. Between these two points, there is an extremely tight bound on the proportion of voxels mutated for any $n > 1$. In this paper, we have treatments Evo, with $n = 2$, and Evo-Devo, with $n = 4$.

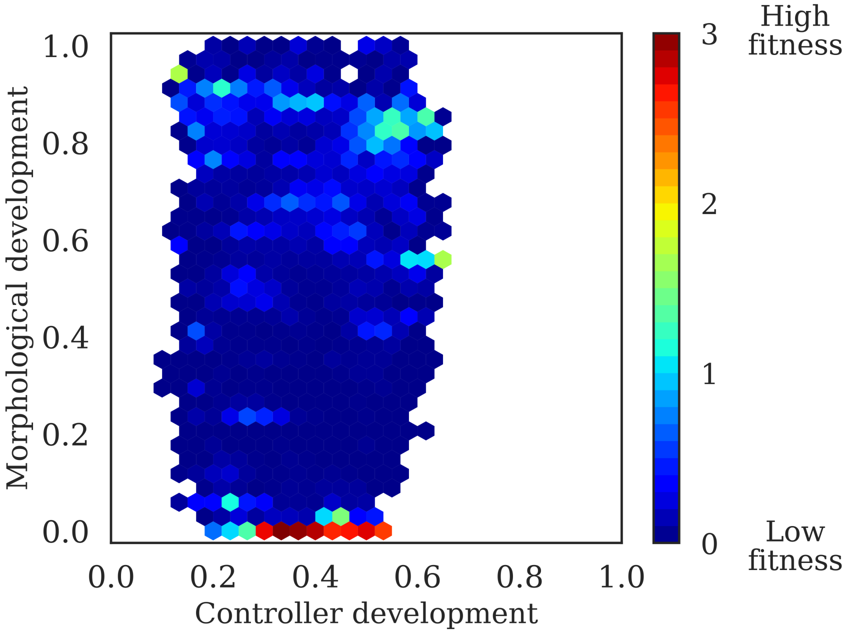

Figure S5. Differential canalization in rigid bodies. This environment consists of a sloped floor, declined toward a light source. Rigid-bodied robots, which are described in Supplementary Methods, perform phototaxis. Although longer legs produce faster walking behaviors, the shortest leg setting places the robot's large, spherical abdomen onto the ground, allowing the robot to simply roll down the ramp toward the light. An ancestor discovers rolling over toward the end of its evaluation period through ontogenetic morphological change that compresses leg lengths. This rollable morphology is assimilated to the start of development through differential canalization: The sooner a robot shrinks its legs, the sooner it can begin rolling; eventually descendants start with compressed legs, are able to immediately roll, exhibit little to no morphological change, but continue to sweep over many different synaptic weights as they behave. Rolling down the slope is not entirely passive, however, since protruding legs, even at their shortest setting, can slow down or even stop forward movement. The best controllers not only avoid such interference, but also guide rolling toward the light source. However, development in this particular case did not affect evolvability. These results are consistent with predictions made by other quantitative models used in theoretical biology that suggest that plasticity only expedites evolution under restrictive conditions[1]. We hypothesize that, in our case, this is because the search space is not deceptive enough: Once the rigid-bodied robot evolves to compress its legs, and touch its abdomen to the sloped floor, it will tend to roll for the remainder of its evaluation period. That is, in contrast with the soft robots, there is no intermediate stage between walking and rolling that suffers the fitness penalty of no longer being able to move.
